# Sleep Treatment Outcome Predictors (STOP) Pilot Study: a protocol for a randomised controlled trial examining predictors of change of insomnia symptoms and associated traits following cognitive–behavioural therapy for insomnia in an unselected sample

**DOI:** 10.1136/bmjopen-2017-017177

**Published:** 2017-12-01

**Authors:** Dan Denis, Thalia C Eley, Fruhling Rijsdijk, Helena M S Zavos, Robert Keers, Colin A Espie, Annemarie I Luik, Isabella Badini, Sarah Derveeuw, Alvin Romero, John Hodsoll, Alice M Gregory

**Affiliations:** 1 Department of Psychiatry, Beth Israel Deaconess Medical Center, Harvard Medical School, Boston, MA, USA; 2 MRC Social, Genetic, and Developmental Psychiatry Centre, Institute of Psychiatry, Psychology, and Neuroscience, King’s College London, London, UK; 3 School of Biological and Chemical Sciences, Queen Mary University of London, London, UK; 4 Sleep and Circadian Neuroscience Institute, Nuffield Department of Clinical Neurosciences, University of Oxford, Oxford, UK; 5 Big Health Ltd, London, UK; 6 Department of Psychology, Goldsmiths, University of London, London, UK; 7 SLaM BioResource for Mental Health, South London and Maudsley NHS Foundation Trust, King’s College London, London, UK; 8 Department of Biostatistics, Institute of Psychiatry, Psychology, and Neuroscience, King’s College London, London, UK

**Keywords:** sleep medicine, psychiatry, adult psychiatry

## Abstract

**Introduction:**

Cognitive–behavioural therapy for insomnia (CBT-I) leads to insomnia symptom improvements in a substantial proportion of patients. However, not everyone responds well to this treatment, and it is unclear what determines individual differences in response. The broader aim of this work is to examine to what extent response to CBT-I is due to genetic and environmental factors. The purpose of this pilot study is to examine feasibility of a design to test hypotheses focusing on an unselected sample, that is, without selection on insomnia complaints, in order to plan a larger behavioural genetics study where most participants will likely not have an insomnia disorder.

**Methods and analysis:**

A two parallel-group randomised controlled trial is being conducted across three London universities. Female students (minimum age 18 years) enrolled on a psychology programme at one of the three sites were invited to participate. The target number of participants to be recruited is 240. Following baseline assessments, participants were randomly allocated to either the treatment group, where they received weekly sessions of digital CBT-I for 6 weeks, or the control group, where they completed an online puzzle each week for 6 weeks. Follow-up assessments have taken place mid-intervention (3 weeks) and end of intervention (6 weeks). A 6-month follow-up assessment will also occur. Primary outcomes will be assessed using descriptive statistics and effect size estimates for intervention effects. Secondary outcomes will be analysed using multivariate generalised estimating equation models.

**Ethics and dissemination:**

The study received ethical approval from the Research Ethics and Integrity subcommittee, Goldsmiths, University of London (application reference: EA 1305). DNA sample collection for the BioResource received ethical approval from the NRES Committee South Central—Oxford (reference number: 15/SC/0388). The results of this work shall be published in peer-reviewed journals.

**Trial registration number:**

NCT03062891; Results.

**Strengths and limitations of this study**

- This study contains a large sample size for a pilot study and will provide valuable effect size information useful for the planning of future investigations of this topic.
- Stratification on sleep problems was implemented to ensure baseline sleep problems were equal in both groups.
- Recruitment was done using convenience sampling, which may lead to some self-selection bias in the sample.
- The online nature of the study makes it difficult to fully assess adherence to the intervention.

## Introduction

Insomnia occurs frequently and causes a substantial burden to society.^1^ It is estimated that as many as one-third of US adults experience issues with their sleep, and the annual cost of insomnia to the US labour force has been estimated to be approximately US$280 billion.^2^ Historically, insomnia has been considered as secondary to other psychiatric disorders, such as depression.^3 4^ More recently, however, it has become clear that insomnia is associated with a wide range of psychiatric conditions and may precede and predict their development and severity.^5–7^

Cognitive–behavioural therapy (CBT) has been shown to be an effective treatment for insomnia.^8 9^ Consequently, the American College of Physicians now recommends CBT for insomnia (CBT-I) as the first-choice treatment for chronic insomnia.^10^ CBT-I is now more accessible than ever due to the development of automated online programmes, which have shown promising effectiveness.^11–13^ A randomised controlled trial of 164 participants meeting diagnostic and statistical manual of mental disorers, 5th edition (DSM-5) criteria for insomnia disorder showed significant post-treatment improvements in insomnia symptoms and sleep efficiency for those participants assigned to a digital CBT-I group compared with a placebo.^11^ These results were largely maintained at an 8-week follow-up. Following this, a meta-analysis of 15 randomised controlled trials investigating digital CBT-I found across all studies a significant improvement in sleep efficiency (7.2%) following digital CBT-I compared with baseline and a significant drop on the Insomnia Severity Index, bringing patients to a subthreshold level of insomnia.^13^ This reduction in insomnia symptom severity was also accompanied by a significant drop in symptoms of depression, suggesting CBT-I may also be effective for problems associated with insomnia.^14^

Despite the demonstrated effectiveness of CBT-I, some individuals fail to respond to treatment. It has been suggested that CBT-I can significantly reduce symptoms of insomnia in around 70% of patients, meaning 30% of patients show no improvement in symptoms.^15^ Understanding the reasons why people either respond or do not respond to treatment holds promise of improving or tailoring current treatments for insomnia.

Investigations of predictors of treatment response to CBT applied to other conditions, such as anxiety, have shown a wide range of demographic, clinical, genetic and epigenetic factors influence response to CBT treatment for anxiety.^16–19^ Genetic predictors of treatment outcome are still unclear however. While some studies have reported specific genetic markers for intervention outcome in disorders such as post-traumatic stress disorder,^20^ panic disorder^21^ and social anxiety disorder,^22^ these findings have not always been replicated.^23 24^ While individually, any genetic predictor is likely to only explain a small proportion of variance in treatment outcome, understanding these multiple factors and their interactions may serve an important role in improving the outcome of therapy. The aim of this pilot study is to test the feasibility of running a larger-scale study of predictors of treatment outcome for CBT-I within a twin design, where not all participants will have insomnia.

## Primary objectives

### Sleep improvement after CBT-I in an unselected sample

To date, studies examining CBT-I have done so in the context of improving symptoms in patients diagnosed with insomnia disorder.^11^ However, we plan to include both participants with and without insomnia in the main study. In particular, as our future study is likely to focus on *twin pairs*, it is inevitable that at least some of the participants in that study will not have insomnia (eg, one participant may have insomnia but their co-twin might not). We are therefore interested to see the extent to which CBT-I has an effect in an unselected sample, that is, participants with and without an insomnia disorder. This pilot aims to establish the distributional properties of individual differences in change score on measures such as the Sleep Condition Indicator (SCI) and Pittsburgh Sleep Quality Index (PSQI)^25 26^ as a result of the CBT-I intervention. The outcome of this will be used to assess the feasibility of running a larger behavioural genetics study in the future investigating genetic predictors of CBT-I outcome in an unselected sample. As we are primarily interested in change in insomnia symptoms, the SCI will be our main outcome measure for this objective (see the Measures section for detail).

### Participation rate and treatment acceptability

The second aim of the study is to assess the feasibility of a digital CBT-I intervention study in a non-clinical group. For example, will participants without insomnia be willing to complete a 6-week online programme aimed at improving sleep? As such, we will be closely monitoring participation (the proportion of participants who are willing to take part in the study) and drop-out rates (the percentage of participants who sign up to the study and drop out before the end of the study). We will also examine treatment acceptability. While psychological interventions to treat insomnia such as CBT-I have been rated highly by patients with regards to how acceptable they find the treatment,^27 28^ it is important for this study to investigate whether participants who do not necessarily have a sleep disorder find CBT-I an acceptable treatment. This will be assessed using an adapted version of the Treatment Acceptability Questionnaire (TAQ)^29^; for more information, see the Measures section.

## Secondary aims

### Factors predicting treatment outcome

We will collect data on factors that may predict treatment outcome and will be able to use the data collected to estimate effect sizes for various predictors, which will be useful in power calculations to estimate the sample size for a larger future behavioural genetics study. Furthermore, by including these measures in the pilot study, it will allow us to assess the feasibility of administering a large battery of questionnaires to participants in addition to completing the digital CBT-I/puzzles.

Based on previous work into predictors of treatment outcome in CBT for anxiety,^16–19^ a wide range of demographic, clinical and genetic predictors such as potential single-nucleotide polymorphisms and polygenic risk scores of response to digital CBT-I will be investigated (see the Measures section for more details) for producing an estimated effect size that will be helpful in the planning of a larger study.

### Sleep quality and implications for associated variables

Sleep quality is known to be associated with a number of other variables, such as anxiety and depression.^30^ As such, one outcome of improving sleep quality through CBT-I could be an improvement of symptoms in associated variables. For example, in a meta-analysis of digital CBT-I randomised controlled trial (RCT) studies, it was found that digital CBT-I significantly reduced depression severity.^14^ As such, digital CBT-I holds the promise of improving sleep problems themselves and the variables commonly associated with them.

Our focus here is to obtain an approximate effect size for any effect that can be used in designing a more substantial study, but we note that we may not have power to report significant effects in the context of this pilot work.

## Exploratory aims of the pilot study

### Genetic predictors of treatment outcome

We will perform preliminary analysis on possible genetic predictors of digital CBT-I treatment outcome to help in the development of hypotheses for a larger genetics study in the future. The samples will be genotyped on the Psychiatric Genomics Consortium customised HumanCoreExome-24 V.1.1 beadchip from Illumina. This beadchip retains a genome-wide association study (GWAS) backbone, exome beadchip content and an additional ~50K psychiatric relevant variants. We will also perform exploratory investigations on the genetic data in relation to exploratory aim 8 (see below).

### Mechanisms mediating improvements in insomnia

Assuming enough variation in appropriate measures, we will investigate whether factors such as pre-sleep arousal, cognitions about sleep, chronotype and specific sleep disturbances mediate CBT-I outcome in an unselected sample.

### Improvement in sleep paralysis episodes following CBT-I

Sleep paralysis is an unusual but relatively common condition involving a period of inability to perform voluntary movements at either sleep onset or on awakening,^31^ with an estimated prevalence of up to 30%.^32 33^ If enough participants with sleep paralysis are included in our sample, we will assess the feasibility and effectiveness of digital CBT-I in the patient groups in terms of reducing the frequency of episodes, as well as associated fear and hallucinations.

### Variables associated with exploding head syndrome

Exploding head syndrome is an unusual experience, characterised by hearing loud noises (eg, an explosion or gunshot) in one’s head at either wake–sleep or sleep–wake transitions.^31^ If enough participants with exploding head syndrome are included in our study, we shall look at potentially associated variables such as insomnia symptoms, stress and psychopathology.

## Methods and analysis

### Study dates

Recruitment for the study started November 2016, and data will have finished being collected by the end of September 2017. The study was retrospectively registered on 5 December 2016. The reason the trial was registered retrospectively was due to very restricted limitations on when participants could be recruited (see the Participant recruitment section). Unfortunately, the trial was not registered until after the first recruitment dates had passed. Rather than lose potential recruiting opportunities, we decided to register the trial retrospectively.

### Design

The study was a two-group parallel randomised controlled trial in which the intervention group received a digital CBT-I intervention and the control group received a weekly online puzzle. See the Intervention section for more details.

**Figure 1:**
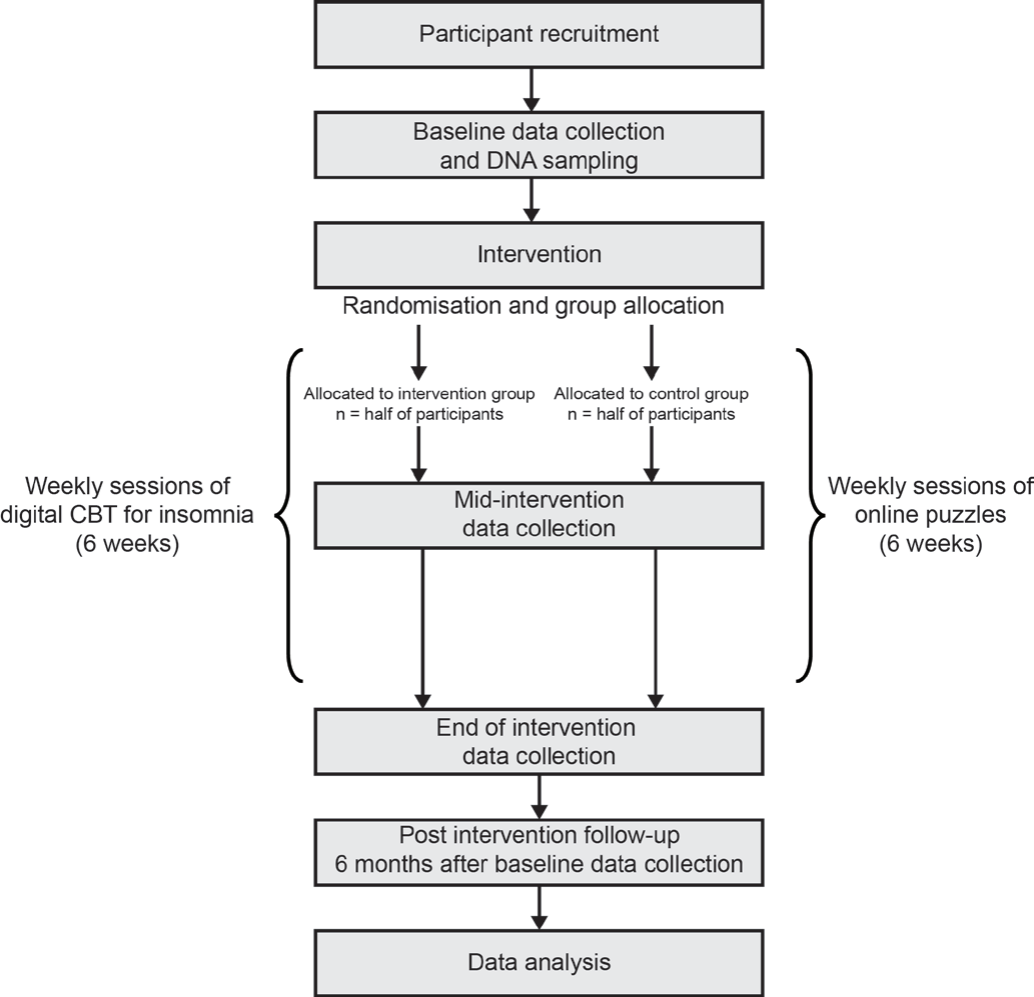
Flow chart of study timeline. CBT, cognitive–behavioural therapy.

Participants were female students (both undergraduate and postgraduate) completing a psychology programme at one of three London universities (for full details, see the trial registration). After completing the baseline assessment online via the Qualtrics system, participants were randomly allocated to either the CBT-I or puzzles group. Three weeks later, participants completed a second online assessment and then a third online assessment 6 weeks after the start of the study. Finally, a follow-up online assessment was carried out 6 months after group allocation. Participants were also invited to give a DNA sample at the start of data collection. While we have limited statistical power to look at genetic predictors of treatment outcomes in the pilot study, these samples could be pooled with other data collected in the future and also provide a useful opportunity for our collaborators to collect data for another ongoing research initiative.^34^ See figure 1 for a detailed outline of the study timeline. A completed Standard Protocol Items: Recommendations for Interventional Trials checklist and WHO trial registration data set can be found in online supplementary files 1 and 2.

### Inclusion and exclusion criteria

Only women were eligible for participation. This is because the majority of the students on the psychology courses are women, and so adding men would create heterogeneity but without sufficient power to examine this further. Furthermore, only individuals enrolled in a psychology course from one of three London universities were recruited due to reasons of convenience. We focused our recruitment efforts on first year students in particular, as it is possible that a small number of students in other years may have already taken part in studies using the same digital CBT-I platform.^35^ In order to address this point explicitly, in the questionnaires given to participants, they were asked if they have had any experience with Sleepio before taking part in this study.

### Participant recruitment

Participants were recruited to the study using a two-step procedure. Initially, potential participants were contacted via an e-mail that provided the study information, specific instructions as to the nature of the recruitment day and contact information.

The second stage of recruitment involved a series of recruitment days at the three sites. These recruitment days were timed to coincide with classes that potential participants were present at, to make it more likely that they would be in university. At sign-up, participants were given a paper copy of the information sheet and were given the option to ask any questions about the nature of the study. After confirming that they were happy to take part in the study, all participants were asked to give informed consent, provide a DNA sample (see the DNA sample collection section) and were assigned a unique participant ID number, which was used for future assessments. To allow the participation of individuals who wished to take part in the study but were unable to sign up in person, participants were given the option to contact the research team directly by email in order to arrange providing consent to take part in the study.

Participants were rewarded for their time, either in the form of course credits (offered credits + £5 online gift voucher) or online shopping voucher (£40), awarded to them on completion of the study.

### Randomisation and study automation

After collection of baseline data, participants were randomly allocated to either the CBT-I group or the puzzles group. A member of the research team randomised eligible participants using the blockrand package for R.^36^ Participants were stratified based on age, sleep problems and study site. Stratification on age was performed to assure similar age distributions in both groups. Stratification on sleep problems was implemented to avoid the possibility of a disproportionate number of participants with sleep problems being randomly allocated to the same group. Stratification for study site was implemented to avoid an unnecessary delay between completing the first questionnaire and being allocated to a group.

An automated email was sent to participants to inform them of which group they had been assigned. Those in the CBT-I group were given further information as to the nature of the programme (see the Digital CBT-I section) as well as a unique code needed to log in to the website. Those in the puzzles group were given information as to the nature of the tasks that they were required to complete (see the Puzzles section). Participants were not able to change groups once they had been allocated.

## Intervention

### Digital CBT-I

CBT-I participants received six weekly CBT-I sessions delivered by an animated ‘virtual therapist’ (The Prof) via the online platform ‘Sleepio’ (http://www.sleepio.com). The programme comprised a fully automated media-rich web application, driven dynamically by baseline, adherence, performance and progress data, and provides additional access to elements such as an online library with background information, a community of fellow users and support, prompts and reminders sent by email.

The Sleepio programme covers behavioural (eg, sleep restriction, stimulus control) and cognitive (eg, putting the day to rest, thought restructuring, imagery, articulatory suppression, paradoxical intention, mindfulness) strategies, as well as additional relaxation strategies (progressive muscle relaxation and autogenic training) and advice on lifestyle and bedroom factors (sleep hygiene). As part of the intervention, participants filled in a daily sleep diary. The intervention was based on a previously validated manual.^37–39^ Sleepio has been shown to improve sleep and associated daytime functioning in adults diagnosed with insomnia disorder.^11^

### Puzzles

Participants in the control group were sent weekly puzzles to complete within Qualtrics. Each puzzle was designed to be cognitively engaging, and time taken to complete a puzzle was matched as closely as possible to the time taken to complete one session of digital CBT-I. Puzzles were sent directly to participants via automated distribution emails sent at 7-day intervals. In order to track whether participants were completing the puzzles, they were required to enter their participant ID number at the start of each puzzle. The types of puzzles administered to participants included word searches, crosswords and lateral thinking problems.

## Data collection

### DNA sample collection

This project was conducted in collaboration with the National Institute for Health Research (NIHR) Biomedical Research Centre (BRC) BioResource for Mental and Neurological Health in London as part of a national NIHR initiative to build up a central library of information (or ‘BioBank)’ about people’s health.

In this study, we obtained saliva samples from our participants after obtaining consent during the recruitment days. Samples were collected by a researcher from the BioResource team in compliance with their ethically approved protocol. The BRC is the custodian of the samples received. On receipt, samples were logged and prepared for extraction of DNA. The BRC ensured that genetic samples were processed in accordance with strict health and safety guidelines and under the requirements of the Human Tissue Act (HTA). King’s College London holds a HTA licence, number: 12293. All samples are stored in tubes labelled with a barcode that includes the participant number. The link between the participant ID and de-identified data is kept in a secure folder. The DNA samples collected as part of this study are stored by the BRC for future analysis and hypothesis testing with appropriate ethical approval in the future, and under existing BRC BioResource approvals.

### Wave 1 data collection

Eligible participants were given the option of completing the baseline survey online after signing up. Participants were encouraged to complete the survey within 1 week from sign-up. Paper copies were made available for participants who had problems with their device or internet access.

Participants completed all measures, as shown in table 1. Participants had the option to leave out any question. The survey took 30–40 min to complete. At the end the survey, participants were reminded that they would be contacted with regards to future data collection.

**Table 1:** Schedule of enrolment, interventions and assessments made at each wave

	Measures	Enrolment	Wave 1 baseline	Allocation	Wave 2 (3 weeks) mid-assessment	Wave 3 (6 weeks) end assessment	Wave 4 (6 months) follow-up
Enrolment							
Eligibility screening		X					
Informed consent		X					
Saliva DNA sample		X					
Allocation				X			
Interventions							
CBT-I					X	X	
Puzzles					X	X	
Assessments							
Demographics			X				
Medical history			X				
Weight and height			X			X	X
Time of year			X		X	X	X
Sleep measures	SCI		X		X	X	X
	PSQI		X		X	X	X
	PSQI-A		X			X	X
	PSAS		X		X	X	X
	DBAS		X		X	X	X
	MCTQ		X			X	X
	WUSEQ		X			X	X
	FISPI		X			X	X
	MUPS		X				
Well-being measures	STAI		X		X	X	X
	MFQ		X		X	X	X
	ADHD		X			X	X
	SPEQ		X			X	X
	PMH		X		X	X	X
	PSS		X		X	X	X
	LTE		X		X	X	X
Lifestyle measures	Sleeping arrangements		X		X	X	X
	Alcohol intake		X		X	X	X
	Caffeine intake		X		X	X	X
	Smoking behaviour		X				
	Vaping behaviour		X				
Treatment acceptability	TAQ*				X	X	

*Only administered to the Sleepio group.

ADHD, attention deficit hyperactivity disorder; CBT-I, cognitive–behavioural therapy for insomnia; DBAS, Dysfunctional Beliefs About Sleep Scale; FISPI, Fearful Isolated Sleep Paralysis Interview; LTE, List of Threatening Experiences; MCTQ, Munich Chronotype Questionnaire; MFQ, Moods and Feelings Questionnaire; MUPS, Munich Parasomnia Screening; PMH, Positive Mental Health Scale; PSAS, Pre-sleep Arousal Scale; PSQI, Pittsburgh Sleep Quality Index; PSQI-A, Pittsburgh Sleep Quality Index Addendum; PSS, Perceived Stress Scale; SCI, Sleep Condition Indicator; SPEQ, Specific Psychotic Experiences Questionnaire (paranoia, hallucinations and cognitive disorganisation subscales); STAI, State–Trait Anxiety Index; TAQ, Treatment Acceptability Questionnaire; WUSEQ, Waterloo Unusual Experiences Scale.

### Waves 2–4 data collection

The second and third waves of data collection were carried out 3 weeks and 6 weeks following allocation. The fourth wave was carried out 6 months following the allocation of participants to groups. These time points corresponded to mid-intervention, end-of-intervention and post-intervention follow-up time points, respectively (see figure 1 for more detail). Automated emails distributed by Qualtrics were sent to participants at the designated intervals. Not all measures are assessed at all waves, as shown in table 1. Follow-up emails to non-responders were sent each week to participants who fall behind on their tasks (ie, CBT-I, puzzles or surveys).

## Measures

Descriptions for all measures used are provided below. For waves 2–4, some measures were adapted to ask participants to consider their answers with reference to the last 2 weeks (unless otherwise stated below) in order to ensure participants were considering only the time since the last wave of data collection when responding. Full details on each measure used can be found in online supplementary file 3.

10.1136/bmjopen-2017-017177.supp3 Supplementary file 3

Demographic information was collected at baseline. At the start of each survey, participants were asked to indicate whether it was currently term time, examination time or holiday time. At wave 2, participants in the Sleepio group indicated whether they had ever used Sleepio before.

### Sleep measures

Insomnia symptoms—*Sleep Condition Indicator*.^25^ An eight-item measure assessing symptoms of insomnia used to identify insomnia symptoms in community samples.^40^

Sleep quality—*Pittsburgh Sleep Quality Index*.^26^ An 18-item questionnaire assessing seven components of sleep quality and disturbances, which also yields a global score of sleep quality. The scale has been shown to be reliable and valid in assessing sleep quality in adult community samples.^41^

Trauma-related sleep disturbances—*Pittsburgh Sleep Quality Index Addendum*.^42^ Assesses frequency of seven sleep disturbances typically related to trauma. The measure has been validated for use in assessing these disturbances.^42 43^

Pre-sleep arousal—*Pre-sleep Arousal Scale*.^44^ Measures symptoms of cognitive (eight items) and somatic (eight items) arousal experienced around bedtime. It is has been validated with respect to objective measures of pre-sleep arousal.^45 46^

Cognitions about sleep—*Dysfunctional Beliefs About Sleep Scale*.^47^ A 10-item questionnaire that includes items about sleep-disruptive cognitions such as faulty beliefs, worry and attentional bias. The measure has shown to be reliable.^48^

Chronotype—*Munich Chronotype Questionnaire* (MCTQ).^49^ Chronotype is estimated as the midpoint of sleep on workdays and work-free days minus half of the difference between sleep duration on work-free days and average sleep duration of the work to control for sleep debt (ie, the midpoint of sleep on work-free days, corrected for sleep duration). The MCTQ is a reliable and valid measure of chronotype.^50 51^

Sleep paralysis—*Waterloo Unusual Experiences Questionnaire* (WUSEQ).^52^ Items from the WUSEQ were used to assess the frequency of sleep paralysis and associated hallucinations. The measure is valid and reliable in healthy student samples.^53 54^

Sleep paralysis—*Fearful Isolated Sleep Paralysis Interview* (FISPI).^55^ Two items from this measure were adapted to measure the amount of fear/distress typically caused by sleep paralysis episodes and how much interference with waking life episodes have caused. The FISPI has been used as a valid and reliable measure of sleep paralysis in university samples.^56^

Exploding head syndrome—*Munich Parasomnia Screening* (MUPS).^57^ Lifetime prevalence of exploding head syndrome was measured using a single item from the MUPS.

### Psychopathology and well-being measures

Anxiety symptoms—*State–Trait Anxiety Index* (STAI).^58^ The STAI assesses both state (20 items) and trait (20 items) levels of anxiety, and is a valid and reliable measure of anxiety symptoms.^59^

Depressed mood—*Mood and Feelings Questionnaire* (MFQ).^60^ Depressed mood was measured using the 13-item MFQ. This has been shown to be a valid measure of depressed mood.

Attention deficit hyperactivity disorder (ADHD) symptomatology—Bespoke measure examined 18 symptoms of ADHD according to DSM-5 criteria.^61^ This is a valid and reliable measure of ADHD symptoms, and has been previously used in young adults to assess ADHD symptomatology in the context of sleep quality.^62^

Psychotic experiences—*Specific Psychotic Experiences Questionnaire*.^63^ Subscales relating to paranoia,^64^ hallucinations^65^ and cognitive disorganisation^66^ were used as they are strongly related with sleep disturbances.^67^ The scale has been shown to have good reliability and validity.^63^

Positive mental health—*Positive Mental Health Scale*.^68^ Positive aspects of health and life experiences were assessed using a nine-item questionnaire.

Life stress—*Perceived Stress Scale* (PSS).^69^ Life stress was measured with a 10-item measure. A review of articles assessing the psychometric properties of the PSS found the measure to be a reliable and valid measure of life stress.^70^

Exposure to threatening events—*List of Threatening Experiences* (LTE).^71 72^ Participants were asked to indicate whether they had experienced any threatening events from a list of 24. The LTE has been shown to have high reliability and be a valid measure of exposure to potentially threatening experiences.^73^

### Lifestyle measures

At each wave, participants were asked about their sleeping arrangements,^74^ and alcohol^75^ and caffeine intake.^76^ Cigarette^75^ and electronic cigarette usage^77–79^ were assessed at baseline.

### Treatment acceptability

The six-item *Treatment Acceptability Questionnaire* (TAQ)^29^ asked specific questions regarding the degree to which they found the treatment acceptable, ethical and effective. There were also specific questions about the nature of the virtual therapist. Only participants in the Sleepio group received the TAQ.

### Sample size

For this study, the target was to have 200 participants, which should provide power to examining our primary research questions, though we plan to over-recruit to account for some attrition throughout the study. As such, 240 participants will be recruited. Power analyses are often conducted using hypothesised effect sizes based on mean differences (eg, before and after treatment). However, as this is a pilot for a future behavioural genetics study, the main statistic of interest is not mean differences but individual differences (ie, variances). The decision to recruit 200 participants for this pilot study was mainly based on personal experiences of recruiting undergraduates from our institutions.

## Statistical analysis

### Primary objectives

#### CBT-I in an unselected sample

The aim is to examine variation in response to CBT-I (ie, variation in the change score of self-reported insomnia symptoms, as measured by the SCI). To this end, we will obtain an effect size for the difference in change scores between the two groups on the SCI scale. Previous RCTs using the SCI as an outcome measure have observed a large effect size (Cohen’s d=1.50) when comparing baseline score with post-treatment score.^11^ It is possible in our sample the effect size will be smaller, given the fact that participants will not necessarily meet insomnia criteria. Nevertheless, a small effect is still expected.

We will then look at differences between groups, by comparing the percentage of participants who finish with SCI scores in different ranges. We will also look at how many participants score below and above the suggested cut-off score for probable insomnia symptoms. Previous data suggest a cut-off of 16, with a score below that meaning probable symptoms of insomnia.^25^ Furthermore, we will calculate the percentage of participants in the digital CBT-I group that will be above the mean score of the control group (Cohen’s U_3_), the percentage of the two groups that overlap and the probability that one person picked at random from the digital CBT-I group will have a higher score than a person picked at random from the control group (the probability of superiority).^80^

#### Participation rate and treatment acceptability

Evaluation of participation rate and treatment acceptability will be based on the descriptive statistics, that is, percentage of participants who sign up to the study and complete it, the percentage from each group who drop out at each stage and mean scores on treatment acceptability questionnaire. Ninety-five per cent CIs for participation rate and acceptability scores will be calculated, which will show the upper and lower bound values of where the true population parameter will appear. Formal tests will be conducted to compare participant rates between the two groups (χ^2^ analysis). For treatment acceptability, χ^2^ tests will test the proportion of participants selecting each response option.

### Secondary objectives

#### Factors predicting treatment outcome and sleep quality and implications for associated variables

We will test which measures at baseline are moderators of longitudinal outcome of change scores in insomnia symptoms with multivariate generalised estimating equation models using Akaike/Bayesian information criteria to select an optimal model with predictors of insomnia symptoms/sleep quality derived. All models will be run in Stata and control for covariates (eg, age) and the non-independence of sibling-pair data. Missing data shall be accounted for using maximum likelihood or multiple imputation procedures. Due to the small sample size, power may not be sufficient to investigate interaction effects. However, they shall be performed as an exploratory analysis.

### Ethics, consent, confidentiality and data security

All stages of the study received ethical approval from the Research Ethics and Integrity subcommittee at Goldsmiths, University of London (application reference: EA 1305). DNA sample collection for the BioResource received ethical approval from the NRES Committee South Central—Oxford (reference number: 15/SC/0388). All participants were asked to provide informed consent before participating. It was made explicit that participation in the study is voluntary, that participants could choose not to answer questions if they did not want to, that they had the right to withdraw from the study at any point and that their data would remain confidential. Participants were informed of the intention to publish results from this study using their data and agreed to this in the informed consent. Copies of questionnaire booklets given to participants are not publically available due to copyright restrictions on some of the measures.

All identifying information was stored in a password-protected document. Survey responses were automatically stored in Qualtrics. No identifying information was stored with response data. Data in Qualtrics are secured using industry best standards (https://www.qualtrics.com/security-statement/). At the end of data collection, datafiles for each wave of the study shall be downloaded off of Qualtric’s servers and stored in SPSS. At this point, the datafiles will be removed from Qualtrics. Only researchers directly involved in the analysis of data will have access to participant data.

### Dissemination of findings

Results of this trial will be disseminated primarily via peer-reviewed journal publications. It is expected that the primary, secondary and exploratory aims 5 and 6 of this study will be reported in a single publication. Other findings of exploratory aims will be published separately. The results of this study will also be available on the ClinicalTrials.gov website when they become available.

### Strengths and weaknesses of the protocol

A key strength of the study was the use of an online CBT-I intervention. The online feature of this intervention is important as it provides easy access for participants. Furthermore, by using an automated system, there was no need for participants to interact with a CBT therapist during the intervention. This made it a more efficient programme than face-to-face CBT, which also meant that it was more feasible to administer to a large sample. It also meant that everyone received the same experience. Examining treatment acceptability for digital CBT-I in a non-selected sample represents a novel investigation that will yield important findings for future researchers wishing to look at this intervention in non-clinical populations. Exploration of potential mechanisms underlying changes in insomnia symptoms is also a strong aspect of this study, as it will contribute to our knowledge of how CBT-I works in reducing symptoms.

Weaknesses include selection bias in the sample. It is possible that those who already suffer from sleep problems (despite not necessarily having an insomnia disorder) were more likely to take part in the study, although recruitment emails emphasised that participants did not have to suffer from any sleep problems to take part. Our convenience sample was also not a representative one, meaning that it may be hard to generalise findings to other populations. Relatedly, it is conceivable that psychology students, as compared with others, may react differently and rate the effectiveness differently to a psychological therapy.

### Judging study success

When considering whether the study will be successful (ie, what results will suggest that a larger, behavioural genetics study is warranted), multiple variables will be considered. These will be the participation rates, treatment acceptability and effect size. As everything will be looked at together, strict criteria will not be set.

10.1136/bmjopen-2017-017177.supp1 Supplementary file 1

10.1136/bmjopen-2017-017177.supp2 Supplementary file 2

## Supplementary Material

Reviewer comments

Author's manuscript
