# Supplementary material for: Sleep Treatment Outcome Predictors (STOP) Pilot Study: a protocol for a randomised controlled trial examining predictors of change of insomnia symptoms and associated traits following cognitive–behavioural therapy for insomnia in an unselected sample
Source: BMJ Open. 2017 Dec 1;7(11):e017177. doi: 10.1136/bmjopen-2017-017177 (PMC5719290; doi:10.1136/bmjopen-2017-017177)
Supplement: Supplementary file 3 [file bmjopen-2017-017177supp003.pdf]

Details of measures

Demographics

Questions were asked regarding the participant's age, date of birth, sex, and ethnicity at the start of the study. They were also asked to report on educational attainment e.g. the number of secondary school education exams taken (General Certificate of Secondary Education (GCSE)/A-levels or international equivalents), and specific grades received during A-level exams or their non-UK equivalent. Participants were also asked to indicate other qualifications received, as well as number of bachelors (e.g. BSc/BA), masters (e.g. MSc), and doctoral degrees (e.g. PhD).

Weight and height

Participants were asked to report their weight either in kilograms or stone. They were also asked to report their height in either centimetres or feet and inches.

Medical history

Participants were asked to rate how good they thought their health was generally, on a 5-point scale ranging from excellent to poor. They were asked whether they had ever been diagnosed by a medical professional with any mood disorders e.g. "*Anxiety or panic disorder*" or nervous system problems e.g. *"Schizophrenia"*. For each illness, they can select either yes or no. If a participant is currently diagnosed with any other psychological or physical illnesses not listed, they were asked to list them.

Use of either prescription medications, and/or use of over the counter, alternative medications, vitamins, minerals, herbal, or other supplements over the last 6 months was assessed by either answering yes, no, or don't know. If a participant has been using any of these, they were asked to name the substances being taken, the reason for use, and whether they took it regularly or occasionally.

Term, exam, or holiday period

At each wave participants were asked: *"At the moment, is it…"*, and chose one of 3 responses. These were: term time, exam time, or holiday time.

Sleep measures

Sleep Condition Indicator (SCI) [1] – In this 8-item questionnaire, participants consider a typical night in the last month and rate various aspects of their sleep including sleep onset, awakenings, perceived sleep quality, the effect of poor sleep on various aspects of life, and the length of their sleep. Higher scores are indicative of better sleep, and scores <= 16 indicate probable insomnia disorder. The SCI is valid, reliable and sensitive to change in insomnia severity [1]. It has been used to identify insomnia symptoms in community samples [2].

Pittsburgh Sleep Quality Index (PSQI) [3] – The PSQI is an 18-item questionnaire assessing 7 components of sleep quality and disturbances (subjective sleep quality, sleep efficiency, sleep disruption, use of sleeping medications, and daytime dysfunction). The scale yields an overall 'global' score of sleep quality, as well as component scores for each of the 7 facets of sleep quality and disturbances assessed, with a higher score indicating greater sleep problems. The

scale has been shown to be reliable and valid in assessing sleep quality in adult community samples [4].

Pittsburgh Sleep Quality Index Addendum (PSQI-A) [5] – Items included in this measure assess sleep disturbances typically related to trauma (disruptive nocturnal behaviours). The frequency of 7 disruptive nocturnal behaviours (e.g. memories or nightmares of a traumatic experience) are assessed with the PSQI-A. The frequency of each disruptive nocturnal behaviour is assessed on a scale ranging from never to 5-7 nights per week, with higher scores indicating a higher frequency of disruptive nocturnal behaviours. The PSQI-A has been shown to be a valid and reliable measure of sleep disturbances related to trauma [5,6].

Pre-sleep Arousal Scale (PSAS) [7] – The PSAS is a 16-item questionnaire measuring symptoms of cognitive (8 items e.g. intrusive thoughts) and somatic (8 items e.g. sweating) arousal experienced around bedtime. Each item is answered on a 5-point scale ranging from not at all to extremely. The scores from each item are summed to create a pre-sleep arousal score for both cognitive (PSAS-C) and somatic (PSAS-S) pre-sleep arousal. For both sub-scales, higher scores indicate greater levels of pre-sleep arousal. The PSAS has been shown to be a valid measure of pre-sleep arousal, and is comparable with objective measures of pre-sleep arousal [8,9].

Dysfunctional Beliefs About Sleep Scale (DBAS-10) [10] – This measure includes questions about sleep-disruptive cognitions such as faulty beliefs, worry, and attentional bias. There are a total of 10 items, and each is answered on a 5-point scale ranging from not at all to extremely. A global score is calculated based on all 10 items, with higher scores indicating higher levels of

dysfunctional beliefs about sleep. Three sub-scales can also be calculated, relating to: 1 – beliefs about the immediate negative consequences of insomnia, 2 – beliefs about the long-term negative consequences of insomnia, and 3 – beliefs about the need to try harder to sleep. The measure has been shown to be a reliable measure of cognitions about sleep [11].

Munich Chronotype Questionnaire (MCTQ) [12] – The MCTQ is a 14-item questionnaire focused primarily on sleep timing, with questions assessing the regularity of one's work schedule, number of workdays per week, sleep timing on workdays and work-free days, and use of an alarm clock on workdays and work-free days. Chronotype is estimated as the midpoint of sleep on workdays and work-free days minus half of the difference between sleep duration on work-free days and average sleep duration of the work to control for sleep debt (i.e. the midpoint of sleep on work-free days, corrected for sleep duration). Importantly, chronotype can only be calculated when individuals do not use an alarm clock on work-free days. The MCTQ is a reliable and valid measure of chronotype [13,14].

Waterloo Unusual Experiences Questionnaire (WUSEQ) [15] – Items for the WUSEQ were used to assess the frequency of sleep paralysis and associated hallucinations. Frequency of sleep paralysis was assessed via the item *"Sometimes when falling asleep or when waking from sleep, I experience a brief period during which I am unable to move, even though I am awake and conscious of my surroundings"*. This is answered on a 7-point scale ranging from never to several times a week. Those who indicated experiencing sleep paralysis at least once are asked follow-up questions about the frequency with which they experience associated hallucinations, on a 4-point scale from never to always. Three categories of sleep paralysis hallucinations are

assessed: intruder, incubus and vestibular-motor [16]. The WUSEQ has been shown to be valid and reliable measure of sleep paralysis frequency and associated hallucinations in healthy student samples [16,17].

Fearful Isolated Sleep Paralysis Interview (FISPI) [18] – Two items from the FISPI were included to assess the amount of fear/distress typically caused by sleep paralysis episodes, and how much interference with waking life sleep paralysis episodes have caused. For each item, participants respond on a scale from none, to very severe. Only participants who report experiencing at least one episode of sleep paralysis will answer these items. The FISPI has been used as a valid and reliable measure of sleep paralysis in university samples [19].

Munich Parasomnia Screening (MUPS) [20] – The lifetime prevalence of exploding head syndrome was measured using a single item from the MUPS. Participants are asked if they had ever noticed the following behaviour: *"When falling asleep or waking, perceiving a loud bang, a sound similar to a bang (e.g. door bang), or having the sensation of an "explosion in the head"".* This item was answered on a scale ranging from never observed, to very frequently (every/almost every night). The MUPS is a valid measure for recognising nocturnal behaviours and parasomnias [20].

Psychopathology and well-being measures

State Trait Anxiety Index (STAI) [21] – The STAI assesses both state and trait levels of anxiety. *State* anxiety was measured using 20 items in which participants were asked to rate the way that

they feel at the present moment (e.g. *"I feel calm", "I am presently worrying over possible misfortunes"*). Each item was assessed on a scale ranging from not at all, to very much so. Higher scores are indicative of greater state anxiety. *Trait* anxiety was measured using 20 items in which participants were asked rate the extent to which they generally feel (e.g., *"I make decisions easily", "I worry too much over something that really doesn't matter"*), on a scale ranging from not at all, to very much so. Higher scores are indicative of greater trait anxiety. The STAI has been shown to be a reliable and valid measure of anxiety symptoms [22].

Mood and Feelings Questionnaire (MFQ) [23] – Depressed mood was measured using the 13-item MFQ. Participants rated the way that they had felt or acted during the past two weeks (e.g. *"I felt miserable and unhappy"*, *"I found it hard to think properly or concentrate"*) on a 3-point scale (not true, sometimes, true). A higher score is indicative of a higher depressed mood over the last 2 weeks. This has been shown to be a valid measure of depressed mood [24].

ADHD – This bespoke measure examined symptoms of attention deficit hyperactivity disorder (ADHD) according to DSM-5 criteria [25]. Participants were asked about 18 symptoms of ADHD according to DSM-5 criteria. Each item is responded as either a yes or no answer. A higher score is indicative of greater ADHD symptomatology. This is a valid and reliable measure of ADHD symptoms, and has been previously used to in young adults to assess ADHD symptomatology in the context of sleep quality [26].

Specific Psychotic Experiences Questionnaire (SPEQ) [27] – The SPEQ measures 6 facets of psychotic experiences. For this study, sub-scales relating to paranoia [28], hallucinations [29],

and cognitive disorganisation [30] were used. These sub-scales were selected based upon prior work showing that these 3 aspects of psychotic experiences are most strongly associated with sleep disturbances in a community sample [31]. As space for questionnaire measures was limited, the 5-highest loading items from each of the 3 sub-scales were selected for inclusion [27]. The paranoia and hallucinations sub-scales were scored on a scale ranging from not at all, to daily, whilst items on the cognitive disorganisation subscale were scored as either yes or no. On all scales, a higher score reflected greater frequency of psychotic experiences. The scale has been shown to have good reliability and validity [27].

Positive Mental Health Scale (PMH) [32] – Positive mental health was assessed using the 9-item PMH. The scale assesses positive aspects of health and life experiences (e.g. *"I am often carefree and in good spirits"*, and *"I am in good physical and emotional condition"*). Each item was answered on a 4-point scale ranging from do not agree to agree. An overall mean score is derived, with a higher score indicating greater positive mental health. The reliability and validity of the measure has been shown to be good [32].

Perceived Stress Scale (PSS) [33] – This 10-item scale was used to measure stress. Participants rated the extent to which they had felt and thought in a certain way over the past month (e.g. *"How often have you felt confident about your ability to handle your personal problems"*), on a scale ranging from never to very often. All items are summed to create an overall score with higher scores indicating a greater level of perceived stress over the past month. A review of articles assessing the psychometric properties of the PSS found the measure to be a reliable and valid measure of life stress [34].

List of Threatening Experiences (LTE) [35,36] – Participants were asked to indicate whether they had experienced any threatening life events from a list of 24. Example events include: *"Been in hospital with a serious illness or injury"*, *"Death of a child or spouse"*, and *"Been sacked from a job"*. For each event, participants were asked to indicate whether they had experienced it recently, answering either yes or no. Scores are summed to create an overall exposure to threatening life events score, with a higher score indicating exposure to a greater number of different threatening events. The LTE has been shown to have high reliability and be a valid measure of exposure to potentially threatening experiences [37].

Lifestyle measures

Sleeping arrangements – Participants were asked 2 questions relating to their sleeping arrangements. They were: 1 – *"During the past month, who usually sleeps in the same room as you"*, and 2: *"During the past month, who usually sleeps in the same bed as you"*. Responses options for both items were: nobody, partner, baby/child, other. These items have been used previous work examining sleep in a healthy sample [38].

Alcohol intake [39] – This was assessed by four items: 1 – *"Do you drink?"* (scored as either yes or no), 2 – *"When you have an alcoholic drink, how many drinks do you have?"* (scale ranged from 1 to 8 or more, with one alcoholic drink being ½ pint of beer or lager/one glass of wine/one glass of spirits/one alcopop), 3 – *"How often do you have an alcoholic drink?"* (scale ranged from once or twice a year to almost every day), and 4 – *"During the last 30 days, how many times did you have five or more alcoholic drinks on the same occasion"* (scale included: four or

more times, three times, twice, once, I have not had five or more drinks on the same occasion in the past month, I have never had five or more drinks on the same occasion). Using items 2 – 4, average alcohol intake in terms of units of alcohol consumed per week can be calculated.

Caffeine intake [40]– The number of caffeinated drinks consumed per day for the last month was assessed. The drinks included were freshly brewed coffee (one shot espresso = one cup), instant coffee, caffeinated tea, and caffeinated soft drinks. The number of each drink type consumed ranged from 0, to 8 or more. The number of each drink type consumed is then recoded to reflect the amount of caffeine present in each type of drink, in order to calculate a score of total caffeine intake.

Smoking behaviour [39] – Three items were used to assess smoking behaviour: 1 – *"Do you smoke?"* (scored as yes, no, or used to but have given up), 2 – *"How often do you smoke cigarettes?"* (scale ranging from every day to once a month), and 3 – *"On the days that I do smoke, I smoke ... cigarettes"* (response options are: 1-5, 6-10, 11-15, 15-20, more than 20). The latter two questions are then used to calculate the number of cigarettes smoked per week.

Vaping behaviour – A bespoke measure of electronic (e) cigarette usage was used based on items from previous studies of e-cigarette usage [41–43]. Usage is assessed using 6 items: 1 – *"Do you use an electronic cigarette (e-cigarette)?"* (scored as either yes or no), 2 – *"How many days have you used an e-cigarette in the past two weeks?"*, 3 – *"Each time you use the e-cigarette, how many puffs do you inhale?"*, 4 – *"What strength of e-cigarette liquid do you use?"* (options are: 0 mg/l, 6 mg/ml, 12 mg/ml, 16mg/ml, 18 mg/ml, 26 mg/ml, other, don't know), 5 – *"How*

many cartridges/refills do you use per day?", and 6 – *"How long does a refill/cartridge typically last (hours)?"*.

Treatment acceptability

Treatment Acceptability Questionnaire (TAQ) [44] – The TAQ is a 6-item measure assessing the treatment acceptability of psychological treatments for adult populations. Participants in the digital CBT-I group are asked the degree to which they find the treatment acceptable, ethical, effective, and likelihood of negative side effects on a 7-point scale, with a lower number indicating lower treatment acceptability. They are also asked two questions specifically about the nature of the therapist, regarding how knowledgeable and trustworthy participants judge them to be. These items are answered using the same 7-point scale, and were adapted for use with a virtual CBT-I therapist used in this study. The measure has been shown to be reliable and valid [44].

References

1 Espie CA, Kyle SD, Hames P, *et al.* The Sleep Condition Indicator: a clinical screening tool to evaluate insomnia disorder. *BMJ Open* 2014;**4**:e004183. doi:10.1136/bmjopen-2013-004183

2 Sheaves B, Porcheret K, Tsanas A. Insomnia, Nightmares, and Chronotype as Markers of Risk for Severe Mental Illness: Results from a Student Population. *Sleep* 2015;:(in press). doi:10.5665/sleep.5342

3 Buysse DJ, Reynolds C, Monk T, *et al.* The Pittsburgh Sleep Quality Index (PSQI): A new instrument for psychiatric practice and research. *Psychiatry Res* 1989;**28**:193–213.

4 Mollayeva T, Thurairajah P, Burton K, *et al.* The Pittsburgh sleep quality index as a screening tool for sleep dysfunction in clinical and non-clinical samples: A systematic review and meta-analysis. *Sleep Med Rev* 2016;**25**:52–73. doi:10.1016/j.smrv.2015.01.009

5 Germain A, Hall M, Krakow B, *et al.* A brief Sleep Scale for Posttraumatic Stress Disorder: Pittsburgh Sleep Quality Index Addendum for PTSD. *J Anxiety Disord* 2005;**19**:233–44. doi:10.1016/j.janxdis.2004.02.001

6 Insana SP, Hall M, Buysse DJ, *et al.* Validation of the Pittsburgh Sleep Quality Index Addendum for Posttraumatic Stress Disorder (PSQI-A) in U.S. Male Military Veterans. *J Trauma Stress* 2013;**26**:192–200. doi:10.1002/jts.21793
7 Nicassio PM, Mendlowitz DR, Fussell JJ, *et al.* The phenomenology of the pre-sleep state: The development of the pre-sleep arousal scale. *Behav Res Ther* 1985;**23**:263–71. doi:10.1016/0005-7967(85)90004-X
8 Broman J-E, Hetta J. Perceived pre-sleep arousal in patients with persistent psychophysiologic and psychiatric insomnia. *Nord J Psychiatry* 1994;**48**:203–7.
9 Robertson JA, Broomfield NM, Espie CA. Prospective comparison of subjective arousal during the pre-sleep period in primary sleep-onset insomnia and normal sleepers. *J Sleep Res* 2007;**16**:230–8. doi:10.1111/j.1365-2869.2007.00579.x
10 Espie CA, Inglis SJ, Harvey L, *et al.* Insomniacs' attributions. *J Psychosom Res* 2000;**48**:141–8. doi:10.1016/S0022-3999(99)00090-2
11 Edinger JD, Wohlgemuth WK. Psychometric comparisons of the standard and abbreviated DBAS-10 versions of the dysfunctional beliefs and attitudes about sleep questionnaire. *Sleep Med* 2001;**2**:493–500. doi:10.1016/S1389-9457(01)00078-8
12 Roenneberg T, Wirz-Justice A, Merrow M. Life between Clocks: Daily Temporal Patterns of Human Chronotypes. *J Biol Rhythms* 2003;**18**:80–90. doi:10.1177/0748730402239679
13 Zavada A, Gordijn MCM, Beersma DGM, *et al.* Comparison of the Munich Chronotype Questionnaire with the Horne-Östberg's Morningness-Eveningness score. *Chronobiol Int* 2005;**22**:267–78.
14 Levandovski R, Sasso E, Hidalgo MP. Chronotype: a review of the advances, limits and applicability of the main instruments used in the literature to assess human phenotype. *Trends psychiatry Psychother* 2013;**35**:3–11. doi:10.1590/S2237-60892013000100002
15 Cheyne JA. Waterloo unusual sleep experiences questionnaire-VIIIa. 2002.
16 Cheyne JA, Newby-Clark IR, Rueffer SD. Relations among hypnagogic and hypnopompic experiences associated with sleep paralysis. *J Sleep Res* 1999;**8**:313–7.
17 Denis D, Poerio GL. Terror and bliss? Commonalities and distinctions between sleep paralysis, lucid dreaming, and their associations with waking life experiences. *J Sleep Res* Published Online First: 2016. doi:10.1111/jsr.12441
18 Sharpless BA, Doghramji K. *Sleep paralysis: Historical, psychological, and medical perspectives*. New York, NY: : Oxford University Press 2015.
19 Sharpless BA. Exploding head syndrome is common in college students. *J Sleep Res* Published Online First: 2015. doi:10.1111/jsr.12292
20 Fulda S, Hornyak M, Müller K, *et al.* Development and validation of the Munich Parasomnia Screening (MUPS): A questionnaire for parasomnias and nocturnal behaviors. *Somnologie* 2008;**12**:56–65. doi:10.1007/s11818-008-0336-x
21 Spielberger C., Gorsuch R., Lushene R, *et al. Manual for the State-Trait Anxiety Inventory*. Palo Alto, CA: : Consulting Psychologists Press 1983.
22 Spielberger CD. *State-Trait Anxiety Inventory: Bibliography*. 2nd ed. Calo Alto, CA: : Consulting Psychologists Press 1989.
23 Angold A, Costello E., Messer S., *et al.* Development of a short questionnaire for use in epidemiological studies of depression in children and adolescents. *Int J Methods Psychiatr Res* 1995;**5**:237–49.
24 Burleson Daviss W, Birmaher B, Melhem NA, *et al.* Criterion validity of the Mood and Feelings Questionnaire for depressive episodes in clinic and non-clinic subjects. *J Child*

Psychol Psychiatry Allied Discip 2006;**47**:927–34. doi:10.1111/j.1469-7610.2006.01646.x
25 American Psychiatric Association. *Diagnostic and Statistical Manual of Mental Health Disorders*. 5th ed. Washington, DC: : American Psychiatric Association 2013.
26 Gregory AM, Agnew-Blais JC, Matthews T, *et al.* ADHD and Sleep Quality: Longitudinal Analyses From Childhood to Early Adulthood in a Twin Cohort. *J Clin Child Adolesc Psychol* 2016;**0**:1–11. doi:10.1080/15374416.2016.1183499
27 Ronald A, Sieradzka D, Cardno AG, *et al.* Characterization of psychotic experiences in adolescence using the specific psychotic experiences questionnaire: Findings from a study of 5000 16-Year-Old Twins. *Schizophr Bull* 2014;**40**:868–77. doi:10.1093/schbul/sbt106
28 Freeman D, Garety PA, Bebbington PE, *et al.* Papers Psychological investigation of the structure of paranoia in a non- clinical population. *Br J Psychiatry* 2005;**186**:427–35. doi:10.1192/bjp.186.5.427
29 Bell V, Halligan PW, Ellis HD. The Cardiff Anomalous Perceptions Scale (CAPS): A new validated measure of anomalous perceptual experience. *Schizophr Bull* 2006;**32**:366–77. doi:10.1093/schbul/sbj014
30 Mason O, Linney Y, Claridge G. Short scales for measuring schizotypy. *Schizophr Res* 2005;**78**:293–6. doi:10.1016/j.schres.2005.06.020
31 Taylor MJ, Gregory AM, Freeman D, *et al.* Do Sleep Disturbances and Psychotic-Like Experiences in Adolescence Share Genetic and Environmental Influences ? *J Abnorm Psychol* 2015;**124**:May 4 [Epub ahead of print]. doi:10.1037/abn0000057
32 Lukat J, Margraf J, Lutz R, *et al.* Psychometric properties of the Positive Mental Health Scale (PMH-scale). *BMC Psychol* 2016;**4**:8. doi:10.1186/s40359-016-0111-x
33 Cohen S, Kamarck T, Mermelstein R. A Global Measure of Perceived Stress. *J Health Soc Behav* 1983;**24**:385–96.
34 Lee EH. Review of the psychometric evidence of the perceived stress scale. *Asian Nurs Res (Korean Soc Nurs Sci)* 2012;**6**:121–7. doi:10.1016/j.anr.2012.08.004
35 Brugha TS, Bebbington P, Tennant C, *et al.* The list of threatening experiences - A subset of 12 life event categories with considerable long-term contextual threat. *Psychol Med* 1985;**15**:189–94.
36 Coddington R. Life events scale for children and adolescents: Measuring the stressfulness of a child’s environment. In: *Stress in Childhood*. New York: : AMS Press 1983.
37 Brugha TS, Cragg D. The List of Threatening Experiences: the reliability and validity of a brief life events questionnaire. *Acta Psychiatr Scand* 1990;**82**:77–81. doi:10.1111/j.1600-0447.1990.tb01360.x
38 McAdams T a., Gregory AM, Rowe R, *et al.* The Genesis 12–19 (G1219) Study: A Twin and Sibling Study of Gene–Environment Interplay and Adolescent Development in the UK. *Twin Res Hum Genet* 2012;**16**:1–10. doi:10.1017/thg.2012.83
39 Currie C, Molcho M, Boyce W, *et al.* Researching health inequalities in adolescents: the development of the Health Behaviour in School-Aged Children (HBSC) family affluence scale. *Soc Sci Med* 2008;**66**:1429–36.
40 Kendler K., Prescott C. Caffeine intake, tolerance, and withdrawal in women: A population-based twin study. *Am J Psychiatry* 1999;**156**:223–8.
41 Foulds J, Veldheer S, Yingst J, *et al.* Development of a questionnaire for assessing dependence on electronic cigarettes among a large sample of ex-smoking e-cigarette users. *Nicotine Tob Res* 2015;**17**:186–92. doi:10.1093/ntr/ntu204
42 Etter JF, Bullen C. Electronic cigarette: Users profile, utilization, satisfaction and

perceived efficacy. *Addiction* 2011;**106**:2017–28. doi:10.1111/j.1360-0443.2011.03505.x
43 Etter JF. Electronic cigarettes: A survey of users. *BMC Public Health* 2010;**10**. doi:10.1186/1471-2458-10-231
44 Hunsley J. Development of the Treatment Acceptability Questionnaire. *J Psychopathol Behav Assess* 1992;**14**:55–64. doi:10.1007/BF00960091
