# Supplementary material for: Sleep Treatment Outcome Predictors (STOP) Pilot Study: a protocol for a randomised controlled trial examining predictors of change of insomnia symptoms and associated traits following cognitive–behavioural therapy for insomnia in an unselected sample
Source: BMJ Open. 2017 Dec 1;7(11):e017177. doi: 10.1136/bmjopen-2017-017177 (PMC5719290; doi:10.1136/bmjopen-2017-017177)
Supplement: Supplementary file 2 [file bmjopen-2017-017177supp002.pdf]

WHO Trial Registration Data Set – The STOP Study

DATA CATEGORY	INFORMATION
Primary registry and trial identifying number	Clinicaltrials.gov. Identifier: NCT03062891
Date of registration in primary registry	5th December 2016
Secondary identifying numbers	NA
Source(s) of monetary or material support	Biomedical Research Centre (BRC) BioResource
Primary sponsor	King's College London
Secondary sponsor(s)	NA
Contact for public queries	Alice M. Gregory<br>Department of Psychology<br>Goldsmiths, University of London<br>London, UK, SE14 6NW<br>Email: a.gregory@gold.ac.uk<br>Telephone: +1 541 346 4075
Contact for scientific queries	Alice M. Gregory<br>Department of Psychology<br>Goldsmiths, University of London<br>London, UK, SE14 6NW<br>Email: a.gregory@gold.ac.uk<br>Telephone: +1 541 346 4075
Public title	The STOP Pilot Study (Sleep Treatment Outcome Predictors)
Scientific title	Sleep Treatment Outcome Predictors (STOP) Pilot Study: A randomised controlled trial examining

DATA CATEGORY	INFORMATION
	predictors of changes of insomnia symptoms and associated traits following cognitive behavioural therapy for insomnia in an unselected sample
Countries of recruitment	United Kingdom
Health condition(s) or problem(s) studied	Symptoms of insomnia
Intervention(s)	Treatment: Online CBT for insomnia (CBT-I). CBT-I participants will receive six weekly sessions delivered by an animated ‘virtual therapist (The Prof) via the online platform ‘Sleepio’. The programme comprises a fully automated media-rich web application, driven dynamically by baseline, adherence, performance and progress data, and provides additional access to elements such as an online library with background information, a community of fellow users, and support, prompts and reminders sent by e-mail.<br><br>Control: Puzzles. Each week participants will be sent a puzzle to complete online (e.g. logic puzzles, crosswords etc.). The puzzles have been designed to be cognitively engaging an take a similar amount of time to one session of Sleepio (20-25 minutes).
Key inclusion and exclusion criteria	Inclusion: Female, aged 18+, psychology student (undergraduate or postgraduate) at one of the three study sites.<br><br>Exclusion: Male, Under 18, not a psychology student at one of the three study sites.
Study type	Interventional<br>Allocation: randomized (stratified by baseline insomnia symptoms)<br>Intervention model: parallel assignment<br>Masking: No masking<br>Primary purpose: treatment
Date of first enrolment	18th November 2016

DATA CATEGORY	INFORMATION
Target sample size	240
Recruitment status	197 participants recruited into the study. Waves 1-3 at various stages of completion. Wave 4 to begin July 2017.
Primary outcome(s)	1. Improvement in sleep problems following online CBT as indicated by changes in insomnia symptoms and subjective sleep quality.<br>2. Assessment of treatment acceptability of the CBT-I in an unselected sample.<br>3. Participation and drop-out rates.
Key secondary outcomes	1. Predictors of response to treatment outcome. Specific predictors being: anxiety, depression, ADHD symptoms, psychotic experiences, positive mental health, stress, and threatening life events. Main statistic of interest will be effect size.<br>2. Improvement in sleep problems through CBT-I to be associated with improvement of symptoms in other variables. Specifically: anxiety, depression, ADHD symptoms, psychotic experiences, positive mental health, stress, and threatening life events. Main statistic of interest will be effect size.
